# Mirror Syndrome (Ballantyne Syndrome): Prenatal Diagnosis, Pathophysiology, and the Role of Fetal Therapy—A Narrative Review

**DOI:** 10.1002/pd.70228

**Published:** 2026-07-30

**Authors:** Riccardo Tudisco, Pauline Latapie, Anita Romiti, Alexandra Benachi, Maelig Abgral, Alexandre J. Vivanti

**Affiliations:** ^1^ Division of Obstetrics and Gynecology “Antoine Béclère” Hospital Paris‐Saclay University Hospitals APHP Clamart France; ^2^ Obstetrics and Obstetrical Pathology Unit Department of Women's and Child Health and Public Health Sciences Fondazione Policlinico Universitario Agostino Gemelli IRCCS Rome Italy

**Keywords:** Ballantyne syndrome, fetal hydrops, mirror syndrome, placentomegaly, preeclampsia, prenatal diagnosis

## Abstract

Mirror syndrome is a rare maternal–fetal condition associated with fetal hydrops and a high risk of adverse maternal and fetal perinatal outcomes. Its diagnosis is challenging due to the lack of standardized diagnostic criteria and its clinical and biochemical overlap with preeclampsia. This narrative review summarizes current evidence on mirror syndrome, with a focus on prenatal diagnosis, placental pathophysiology and the role of targeted fetal therapy. Mirror syndrome is most frequently associated with non‐immune causes of fetal hydrops and available evidence supports a central role for the placenta in disease pathogenesis. Recent data suggest that angiogenic biomarkers, including sFlt‐1 and PlGF, may be useful in the differential diagnosis from preeclampsia. Resolution of fetal hydrops through in utero therapy can lead to rapid maternal improvement and pregnancy prolongation in selected cases. Early recognition and multidisciplinary management are essential to optimize maternal and fetal outcomes.

## Summary

- What is already known about this topic and what does this review add?
  ◦ This review integrates recent evidence supporting a central placenta‐mediated pathophysiology, highlights the emerging diagnostic role of angiogenic biomarkers in differentiating mirror syndrome from preeclampsia, and summarizes contemporary data demonstrating that successful targeted fetal therapy may reverse maternal disease and allow pregnancy prolongation in selected cases.

## 1 Introduction

Mirror syndrome is a rare and potentially severe maternal–fetal condition that may complicate approximately 5%–30% of pregnancies affected by fetal hydrops [1]. Fetal hydrops is uncommon, affecting about 0.02%–0.06% of pregnancies. The true incidence of mirror syndrome in the general obstetric population remains uncertain, as evidence is derived mostly from case reports and case series [2].

The rarity of the condition and inconsistent diagnostic definitions contribute to delayed recognition. Mirror syndrome shares a strong clinical and pathophysiological resemblance to pre‐eclampsia [3, 4]. Clinical management largely depends on the etiology of fetal–placental hydrops [4].

Mirror syndrome remains incompletely characterized, with limited understanding of the mechanisms linking fetal hydrops, placental dysfunction and maternal disease. This review aims to provide a clinically oriented overview of current evidence on the pathophysiology of mirror syndrome and the potential impact of fetal hydrops treatment on maternal disease progression and management.

## 2 Methodology

This article is a narrative review that aims to summarize current evidence regarding the prenatal diagnosis, pathophysiology, clinical presentation, and management of mirror syndrome. A literature search was performed using the PubMed/MEDLINE and Embase databases to identify eligible publications from January 1980 to December 2025.

The search strategy combined Medical Subject Headings (MeSH) terms and keywords including “mirror syndrome,” “Ballantyne syndrome,” “maternal mirror syndrome,” and “fetal hydrops.” The search terms were combined using Boolean operators (AND, OR) as appropriate.

The predefined search strategy retrieved 146 records in PubMed/MEDLINE and 154 records in Embase.

**FIGURE 1:**
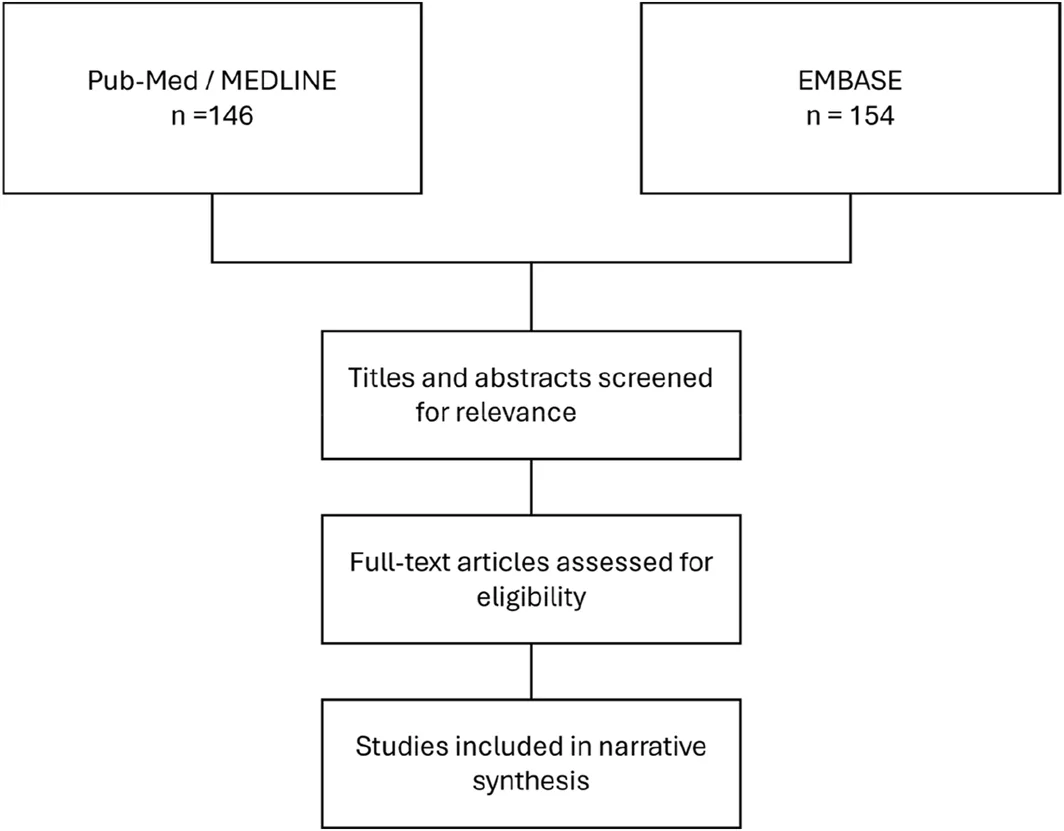
Literature search strategy and study selection process for the narrative review.

Titles and abstracts were screened for relevance, followed by full‐text assessment. Eligible publications included original research articles, observational studies, case series, case reports, and systematic reviews. Given the rarity of the condition, case reports and small retrospective series were considered appropriate sources of evidence. Since this manuscript was designed as a narrative review, a formal PRISMA‐guided study selection process and risk‐of‐bias assessment were not performed. Study selection was performed by the authors based on relevance to the objectives of this narrative review. Although this was a narrative review and not a systematic review, a schematic flow diagram summarizing the literature search and study selection process is provided (Figure 1).

## 3 Definition and Diagnostic Criteria

Mirror syndrome, also termed “triple edema syndrome,” is characterized by three core components:

1. *Fetal component*: Hydrops fetalis, defined as abnormal fluid accumulation in at least two fetal compartments (e.g., ascites, pleural effusion, pericardial effusion, skin edema).
2. *Placental component*: Placentomegaly or placental edema, commonly defined as placental thickness ≥ 40 mm in the second trimester or ≥ 60 mm in the third trimester [5, 6].
3. *Maternal component*: Maternal edema, variably defined as rapid weight gain, generalized or pulmonary edema and/or clinical features suggestive of preeclampsia, including hypertension and proteinuria.

Mirror syndrome most commonly manifests between 16 and 34 weeks of gestation, although cases have been documented as late as 39 weeks, as illustrated by Allarakia et al. [7] Diagnosis often relies on the simultaneous identification of maternal and fetal manifestations, but several reports indicate that fetal signs may precede maternal findings, and vice versa [1]. In the systematic review by Allarakia et al. [7] only 46% of cases presented with simultaneous maternal and fetal manifestations, whereas in 36% of cases fetal abnormalities were detected earlier. The interval between initial maternal and/or fetal manifestations and the diagnosis of mirror syndrome is often short but variable, ranging from a median of 2–11.5 days across published series (Table 1).

**TABLE 1:** Median number of days between the onset of initial maternal and/or fetal manifestations and diagnosis of mirror syndrome.

Study	Median diagnostic latency from symptom onset (days)	Median gestational age at diagnosis [range] (weeks)
Biswas et al. [7]	8	27.1 [16.6–34.4]
Sichitiu et al. [4]	2	27.0^a^ [21–34]
Allakaria et al. [8]	11	27 [16–39]
Braun et al. [9]	NR	NR^b^ [22.5–27.8]
Hirata et al. [10]	11.5	32 [26–39]
Mogharbel et al. [11]	NR^c^	22.4 [16.4–28.7]

Abbreviation: NR, Not Reported.

^a^ Mean.

^b^ Median not reported, only the range was available.

^c^ Not explicitly reported but median gestational age of hydrop diagnosis 19 w + 3 d and median gestational age at diagnosis of mirror syndrome 22 + 3.

The literature on mirror syndrome consists largely of case reports and small retrospective case series with considerable variability in the definition of the syndrome across studies [8, 9, 10, 11, 12, 13]. The adoption of pragmatic diagnostic criteria could be useful to facilitate earlier identification of mirror syndrome in current clinical practice and allow comparisons across studies.

### 3.1 Fetal Component

Fetal hydrops is usually defined as pathological fluid accumulation in at least 2 different fetal compartments, but definitions vary across studies. For example, in the study of Hirata et al. [14], it was defined as fetal edema (fluid beneath the skin at a depth of more than 5 mm) associated with fetal ascites or pleural effusion.

Gestational age at the onset of fetal hydrops may influence the risk of progression to mirror syndrome although data remain inconsistent. According to some studies, cases of fetal hydrops tend to be diagnosed earlier when associated with mirror syndrome than when occurring independently. In the study by Han et al. [9], the median gestational age at the onset of fetal hydrops was significantly later in mirror syndrome cases (27.8 vs. 23 weeks, *p* = 0.006), with an odds ratio of 15.83 for the development of mirror syndrome when fetal hydrops occurred after 24 weeks. In the study of Hirata et al. [14] fetal hydrops occurred earlier in the mirror group (29 vs. 31, *p* = 0.011). The impact of the timing of onset of fetal hydrops over the risk of mirror syndrome was not confirmed in a much larger cohort of 182 cases of hydrops published by Burnett et al. [6] Differences in gestational age at diagnosis and in the reported risk of progression to mirror syndrome among studies may, at least in part, be explained by heterogeneity in etiological factors and study populations.

### 3.2 Placental Component

Placental thickening has been reported as a potential predictive feature for the progression from fetal hydrops to mirror syndrome. However, the definition of placentomegaly varies among studies, ranging from qualitative descriptions to different sonographic thickness cut‐offs [4, 9, 15]. In the largest cohorts, such as those reported by Sichitiu et al. and Burnett et al. [4, 6], explicit sonographic thresholds of placental thickness were applied (≥ 40 mm in the second trimester or ≥ 60 mm in the third trimester).

In their study, Burnett et al. reported that placental thickening was associated with an approximately seven‐fold increased risk of progression to mirror syndrome.

Hirata et al. [14] reported placental edema in 100% of mirror syndrome cases, compared with 71% of hydrops fetalis cases without mirror syndrome. Likewise, in the systematic review by Allarakia et al. [7], among 113 cases of mirror syndrome, placental edema was reported in approximately 63% of cases.

Similarly, in the retrospective series by Chen et al. [12], placental thickness was greater in patients who developed mirror syndrome. Han et al. [9] also identified placental thickening as a risk factor associated with this condition.

### 3.3 Maternal Component

The maternal presentation of mirror syndrome is clinically the richest [3, 4, 7, 8, 15, 16]. As a consequence, the definition of the maternal component of mirror syndrome is the least uniform across studies.

The most commonly reported maternal manifestations include:

- Rapid weight gain secondary to generalized edema
- Hypertension
- Laboratory abnormalities resembling those seen in pre‐eclampsia

Some authors, such as Gedikbasi et al. [17] even consider pre‐eclampsia to be a necessary maternal criterion for the diagnosis of mirror syndrome. The most severe maternal complications include acute pulmonary edema, cardiac failure, and, more rarely, seizures [18, 19].

Among the laboratory abnormalities, the most consistently described finding is maternal hemodilution, with low hematocrit/hemoglobin and hypoalbuminemia (Table 2). Other laboratory findings are not reported in all the series or data are very limited. Liver enzymes may be elevated in some cases of mirror syndrome, but this is not a distinguishing feature. A small number of cases have been reported with clinical and laboratory features overlapping those of HELLP syndrome [8].

**TABLE 2:** Laboratory findings in mirror syndrome.

Laboratory parameter	Finding	Reported differences in MS group versus fetal hydrops without maternal involvement	Evidence
Hematocrit/Hemoglobin	Decreased	Lower	Han et al. [12], Hirata et al. [10], Allarakia et al. [8], Mogharbel et al. [11]
Albumin	Hypoalbuminemia (cut‐off varies between the studies)	Lower	Mogharbel et al. [11], Han et al. [12], Hirata et al. [10]
Uric acid	Increased	Higher	Han et al. [12], Mogharbel et al. [11]
Platelet count	Decreased/normal possible overlap with hemodilution	Lower in some case‐control analyses, but often within normal range [12] No differences in other reports [10, 11]	Mogharbel et al. [11], Han et al. [12], Hirata et al. [10]
Serum creatinine	Mildly increased	Higher	Hirata et al. [10], Han et al. [12]
Liver enzymes (AST/ALT)	Normal/mildly increased	No significant difference	Mogharbel et al. [11], Han et al. [12], Hirata et al. [10]
β‐hCG	Increased	Comparative data are limited. Higher in the cohort of Hirata et al.	Hirata et al. [10]

Some case‐control studies suggest that these abnormalities are more pronounced in mirror syndrome than in fetal hydrops without maternal involvement.

More recently, some authors such as Espinoza et al. [10] have explored the possibility of using maternal biomarkers in the context of suspicion of mirror syndrome, which will be discussed further in the review.

Mirror syndrome is associated with a heterogeneous range of maternal clinical manifestations, as reported in Table 3.

**TABLE 3:** Clinical spectrum of maternal manifestations in mirror syndrome.

Clinical category	Typical manifestations	Approximate frequency^a^
Edematous features	Generalized edema, rapid weight gain	Common (∼60%)
Biochemical abnormalities	Hypoalbuminemia, anemia, hemodilution	Common (∼40%–55%)
Hypertensive features	New‐onset hypertension, proteinuria	Moderate (∼25%–40%)
Respiratory involvement	Pulmonary edema, pleural effusion	Uncommon (∼10%–15%)
Severe maternal complications	HELLP syndrome, cardiac failure	Rare (< 5%)

*Note:* Data derived from Biswas et al. [7].

^a^ Mean.

Despite the substantial heterogeneity in diagnostic definitions across studies, the association of fetal hydrops and maternal clinical signs of fluid overload represents the most consistently reported diagnostic presentation of mirror syndrome. Placentomegaly, hemodilution, hypertensive features, and angiogenic biomarker imbalance may be considered rather than mandatory diagnostic criteria.

## 4 Differential Diagnosis From Preeclampsia

Pre‐eclampsia is a maternal disorder of placental origin, for which delivery remains the only curative treatment. Although certain pathophysiological aspects remain unclear, numerous studies have compared and contrasted pre‐eclampsia and mirror syndrome (Table 4). Biswas et al. [8] reported pre‐eclampsia in 50% of mirror syndrome cases. Despite their distinct nature and uncertain underlying mechanisms, a pathophysiological continuum between the two conditions has been proposed. One of the most consistently reported laboratory findings distinguishing mirror syndrome from pre‐eclampsia is maternal hemodilution, whereas pre‐eclampsia tends to be associated with hemoconcentration [2, 4, 7, 8, 14, 15, 16]. Mogharbel et al. [15] found that hemodilution was present in 100% of mirror syndrome cases and hypertension in 90%. Allarakia et al. [7] also reported the presence of diminished deep tendon reflexes in mirror syndrome, in contrast to the hyperreflexia commonly observed in pre‐eclampsia. Clinical and laboratory findings common to both conditions include headache, edema with rapid weight gain, hypertension, anemia, hyperuricemia, proteinuria, oliguria, and acute pulmonary edema [4, 16, 20].

**TABLE 4:** Key clinical, laboratory and ultrasound differences between pre‐eclampsia and mirror syndrome.

	Preeclampsia	Mirror syndrome
Clinical characteristics		
Edema	Mild/moderate	Severe/generalized
Hypertension	Always present	Possible (∼30%) Not diagnostic
Neurological signs	Hyperreflexia Clonus may occur, especially in severe disease	Typically absent
Laboratory		
Hematocrit	Hemoconcentration	Hemodilution, relative anemia
Platelet count	May be reduced in severe disease	Normal or mildly reduced
HELLP syndrome	Possible	Rarely reported
Proteinuria	Often present. Diagnostic criteria	May be present
Uric acid	Increased	Increased, may be markedly elevated
Serum albumin level	Normal	Decreased
SFlt‐1/PlGF ratio	Markedly elevated (often > 85)	Variable, usually < 85
Ultrasound findings		
Fetus	Fetal growth restriction common	Fetal hydrops
Placental thickness	Normal	Increased
Histological characteristics		
Placental weight and size	Normal or reduced	Increased
Vascularization	Impaired angiogenesis; reduced villous branching	Pronounced neovascularization
Maternal vascular malperfusion lesions	Present	Not typically described
Management and outcomes		
Fetal interventions	Limited role	Indicated
Postpartum course	Usually improves after delivery	Rapid improvement after hydrops resolution or delivery
Maternal outcomes	Increased risk of severe maternal complication	Severe complications uncommon but possible
Perinatal outcome	Adverse outcomes mainly related to fetal growth restriction and iatrogenic prematurity	Depends on hydrops etiology

Fetal ultrasonography plays a central role in diagnostic differentiation. The detection of fetal hydrops should prompt clinical and laboratory evaluation for mirror syndrome. Conversely, pre‐eclampsia is more frequently associated with fetal growth restriction and oligohydramnios.

In conclusion, from a clinical point of view, mirror syndrome should be suspected in cases presenting maternal edema or preeclampsia‐like features in association with fetal hydrops and placental thickening. Pre‐eclampsia should be primarily considered when maternal hypertension and proteinuria occur in the absence of fetal hydrops and are associated with fetal growth restriction or placental insufficiency rather than placental enlargement.

Accurate differential diagnosis has major implications for management because successful treatment of fetal hydrops may reverse maternal disease and improve maternal–fetal outcomes [4], supporting the concept that mirror syndrome is a distinct placenta‐mediated disorder rather than simply a variant of pre‐eclampsia.

## 5 Etiology

Mirror syndrome was initially associated with Rh alloimmunization [20]. With the implementation of routine anti‐D immunoprophylaxis, the incidence of immune hydrops has declined and non‐immune hydrops fetalis accounts for almost 90% of cases [21]. Nevertheless, the immune etiology still accounts for approximately one‐third of reported cases of mirror syndrome [16].

Mirror syndrome has been described in association with a broad range of conditions resulting in fetal hydrops, suggesting that virtually any etiology of fetal anasarca may trigger its development. Reported associations include infectious causes such as parvovirus B19 [22], fetal anemia due to hemoglobinopathies (e.g., alpha‐thalassemia), placental tumors such as chorioangiomas [23], complications of multiple gestation including twin‐to‐twin transfusion syndrome (TTTS), chromosomal abnormalities [24], sacrococcygeal teratoma [25], and CHAOS syndrome [26].

However, accumulating evidence indicates that specific etiologies are more strongly correlated with the occurrence of mirror syndrome. A recent study by Burnett et al. [6] involving a large retrospective cohort of 182 cases of fetal hydrops reported that cardiogenic etiologies were associated with a higher risk of progression to mirror syndrome compared with non‐cardiogenic hydrops.

## 6 Pathophysiology

Although the pathophysiology of mirror syndrome remains unclear, available evidence supports a central role of the placenta. Similar to preeclampsia, placental dysfunction is associated with an imbalance of angiogenic and antiangiogenic factors (as reported by Hobson et al. [3]) and the rapid resolution of maternal manifestations after delivery strongly supports this pathogenic mechanism.

In a case of mirror syndrome secondary to TTTS, Hobson et al. [3] investigated the concentrations of several biomarkers typically elevated in pre‐eclampsia. They showed an increase in inflammatory mediators such as sFlt‐1, ET‐1, sICAM‐1, and vWF in patients presenting mirror syndrome and a marked decrease in the circulating levels of these markers immediately after delivery. Similarly, Espinoza et al. [10] reported higher plasma concentrations of sVEGFR‐1 in patients with mirror syndrome than in those with uncomplicated pregnancies. Villous trophoblastic hypoxia has been proposed as a potential cause of the increased production and release of sVEGFR‐1 into the maternal circulation, contributing to endothelial dysfunction and maternal edema. According to the authors, several factors may contribute to the development of this condition, including the severity of placental edema and genetic determinants influencing the production, metabolism, and functional regulation of pro‐ and anti‐angiogenic factors.

Several clinical reports have also demonstrated elevated serum levels of sFlt‐1 and an increased sFlt‐1/PlGF ratio, mirroring the biochemical profile observed in pre‐eclampsia. However, some authors [7] have suggested that an sFlt‐1/PlGF ratio below 85 could be more indicative of mirror syndrome rather than of pre‐eclampsia. The enhanced secretion of PlGF by the enlarged placental mass has been proposed as a possible explanation for this observation. Conversely, the simultaneous elevation of both the sFlt‐1/PlGF ratio and hCG levels appears to be more characteristic of mirror syndrome [27]. However, the angiogenic imbalance could represent either a primary pathogenic driver or a consequence of placental hydrops, and partial overlap with pathological pathways of pre‐eclampsia cannot be excluded.

Hyperplacentation in mirror syndrome can be demonstrated by elevated levels of human chorionic gonadotropin (hCG) in the maternal circulation. High levels of maternal hCG have been reported in several clinical reports of mirror syndrome [22, 28]. In the retrospective cohort of Hirata et al. [14] maternal hCG levels were significantly higher in the mirror syndrome group, with elevated concentrations observed in 100% of these patients, compared to only 33% in the hydrops fetalis‐only group. Similarly, Chiaki et al. [29] analyzed 16 pregnancies complicated by fetal hydrops and pleural effusion treated with thoraco‐amniotic shunting and showed significantly higher *β*‐hCG levels in the mirror syndrome group, which decreased if the shunting procedure was effective.

Knowing the role of hCG in the pathophysiology of the hydatidiform mole and in inducing increased vascular permeability in ovarian hyperstimulation syndrome, it is possible that hCG may contribute both to the hydropic placental changes observed in mirror syndrome and to the exacerbation of maternal edema [27]. The pathogenic role of hCG is further supported by the rapid decrease in hCG levels following successful laser treatment, as in the case report of Matsubara et al. [30] Occasional reports have described spontaneous ovarian hyperstimulation or hyperreactio luteinalis in pregnancies complicated by mirror syndrome, possibly related to markedly elevated maternal hCG levels [31, 32].

Nevertheless, the pathogenic contribution of hCG remains speculative, and interindividual variability in placental response may partly explain why mirror syndrome develops only in a subset of pregnancies complicated by fetal hydrops [10].

## 7 Histopathology

Several studies have investigated the placental morphology associated with fetal hydrops. Espinoza et al. [10] compared histopathological placental sections from pregnancies complicated by mirror syndrome with those from preterm deliveries. In mirror syndrome, placentas showed immature villi exhibiting extensive edema, numerous syncytiotrophoblastic aggregates, increased intervillous fibrin deposition, and multiple foci of calcification. Such findings were further supported by Graham et al. [2], who reported histological and immunohistochemical findings in placentas from mirror syndrome. In placentas from cases of mirror syndrome, they observed an intact trophoblastic layer, no increase in trophoblastic proliferation, enhanced apoptosis of syncytiotrophoblasts and deposits of anti‐angiogenic factors including sFlt‐1 and soluble endoglin.

In the study by Hobson et al. [3] reporting a case of mirror syndrome occurring after fetoscopic laser photocoagulation for severe TTTS, histological analysis showed that the placenta from the former recipient twin exhibited markedly edematous and congested villi with pronounced neovascularization, while the donor twin's placental territory displayed villous edema, constituting one component of the hydropic triad (mother–fetus–placenta). Finally, Gherman et al. [33] demonstrated a markedly increased placental‐to‐fetal weight ratio. Histological analysis also revealed trophoblastic immaturity, villous edema, and poor cytotrophoblastic migration.

## 8 Specific Management of Fetal Hydrops

The management of mirror syndrome largely depends on the treatment of the underlying cause of fetal hydrops. In their systematic review, Allarakia et al. found no correlation between the etiology of fetal hydrops and fetal or maternal outcomes [7]. Moreover, fetal prognosis did not appear to be influenced by gestational age at the time of mirror syndrome diagnosis, or by the sequence of onset of maternal and fetal symptoms. In contrast, a more recent cohort study by Burnett et al. reported that cardiogenic fetal hydrops was significantly associated with the development of mirror syndrome, suggesting that the underlying etiology may influence maternal risk of mirror syndrome.

Multiple case reports and series have demonstrated improved fetal and maternal outcomes when targeted fetal therapy successfully resolves hydrops. Reported interventions include:

— Surgical interventions in cases of sacrococcygeal teratoma, such as interstitial laser ablation or radiofrequency ablation [25, 34].
— Pleuro‐amniotic shunting [14, 34] in cases of compressive unilateral or bilateral fetal pleural effusion.
— Intrauterine transfusions for hydrops secondary to alloimmunization or parvovirus B19 infection [4, 35].

Trad et al. [1] highlighted the crucial role of fetal therapy, as did Chimenea et al. [35], who through a case series, explored its therapeutic impact on both fetal hydrops and mirror syndrome. The treatment of choice depends on the underlying etiology of fetal edema. In a cohort study published in 2019, Gavin et al. [36] reported that in utero therapy (when feasible) resulted in the resolution of hydrops in 85% of cases, along with improvement in maternal clinical and biochemical abnormalities. Deliveries in this series occurred for reasons unrelated to hydrops or to the progression of mirror syndrome itself. More recently, Sichitiu et al. [4] confirmed the value of fetal therapy, showing that resolution of fetal hydrops may be accompanied by improvement in maternal clinical condition. In their cohort, pregnancy prolongation was achieved in more than one‐third of the cases, with a mean delay of 42 days.

Nevertheless, even after apparent resolution of fetal hydrops, close clinical monitoring of patients remains essential since cases of mirror syndrome onset have been reported up to 7 days after successful treatment of fetal hydrothorax [37]. In the study by Sichitiu et al. [4], mirror syndrome was identified only after fetal therapy in approximately 66% of cases.

## 9 Outcomes

### 9.1 Maternal Outcomes

No maternal deaths have been reported in the literature, but maternal morbidity remains significant. According to the systematic review by Biswas et al. [8], up to 90% of patients experienced one or more potentially life‐threatening complications, including postpartum hemorrhage, intensive care unit (ICU) admission, cardiac failure, acute pulmonary edema, and renal insufficiency. Postpartum hemorrhage was the most frequent complication, occurring in 45% of cases. Rates of ICU admission can reach 50%, primarily due to acute heart failure, disseminated intravascular coagulation, or acute pulmonary edema [11]. The median time to resolution of maternal symptoms ranged from 2 to 13.5 days [8, 9, 16].

### 9.2 Fetal Outcomes

The fetal prognosis in mirror syndrome is generally poor. In the review by Biswas et al. [8], the live birth rate was 7.7%, after excluding pregnancies terminated for medical reasons. Allarakia et al. [7] reported an intrauterine fetal death (IUFD) of 41% among 107 pregnancies complicated by mirror syndrome. Braun et al. [16] reported a mean combined rate of IUFD and neonatal death of 35.7%. Similarly, Sichitiu et al. [4] found 55% live births, 25% neonatal deaths, and 18% IUFD, corresponding to an overall perinatal mortality rate of 44%.

Overall, mirror syndrome appears to be primarily driven by fetal hydrops. Therefore, when feasible, management should focus on treating the underlying cause. When no effective intrauterine therapy is available, management options should be carefully discussed with the patient. Given the poor fetal and neonatal prognosis, an individualized management strategy—including consideration of delivery or pregnancy termination, where legally permitted—should be discussed, with the aim of minimizing both fetal mortality and maternal morbidity.

## 10 Conclusions

This review provides an updated clinically oriented synthesis of the current knowledge on mirror syndrome. It highlights aspects often fragmented across previous studies. Unlike prior reviews that focused on a single aspect of mirror syndrome, we gathered the information on this rare syndrome to allow a better comprehension of the argument, reporting data on diagnostic criteria, pathophysiology, maternal and fetal outcomes, and the role of targeted fetal therapies in a single framework. In cases of mirror syndrome, early recognition, multidisciplinary care, and tailored fetal therapy are critical to improve maternal and neonatal prognosis.

Research perspectives in mirror syndrome are numerous, but strongly limited by the rarity of the condition itself. The integration of exome sequencing and angiogenic biomarkers into clinical practice could potentially allow for a more precise etiological characterization of unexplained cases of fetal hydrops, as well as a deeper understanding of the pathophysiology of mirror syndrome. Given its very low incidence, the establishment of international registries compiling clinical, ultrasonographic, and histological data would represent a crucial step toward defining optimal management strategies and improving prognostic counseling for affected families.

## Funding

The authors have nothing to report.

## Ethics Statement

The authors have nothing to report.

## Consent

The authors have nothing to report.

## Conflicts of Interest

The authors declare no conflicts of interest.

## Data Availability

Data sharing is not applicable to this article as no new data were created or analyzed in this study.

## References

[1] A. Teles Abrao Trad , R. Czeresnia , A. Elrefaei , E. R. Ibirogba , K. Narang , and R. Ruano , “What Do We Know About the Diagnosis and Management of Mirror Syndrome?,” Journal of Maternal‐Fetal and Neonatal Medicine 35, no. 20 (2022): 4022–4027, 10.1080/14767058.2020.1844656. 33722118

[2] N. Graham , A. Garrod , P. Bullen , and A. E. P. Heazell , “Placental Expression of Anti‐Angiogenic Proteins in Mirror Syndrome: A Case Report,” Placenta 33, no. 6 (2012): 528–531, 10.1016/j.placenta.2012.02.016. 22401877

[3] S. R. Hobson , E. M. Wallace , Y. F. Chan , A. G. Edwards , M. W. T. Teoh , and A. P. L. Khaw , “Mirroring Preeclampsia: The Molecular Basis of Ballantyne Syndrome,” Journal of Maternal‐Fetal and Neonatal Medicine 33, no. 5 (2020): 768–773, 10.1080/14767058.2018.1500550. 30614331

[4] J. Sichitiu , F. Alkazaleh , R. De Heus , et al., “Maternal ‘Mirror’ Syndrome: Evaluating the Benefits of Fetal Therapy,” Prenatal Diagnosis 44, no. 8 (2024): 979–987, 10.1002/pd.6589. 38752664

[5] R. Strebeck , B. Jensen , and E. F. Magann , “Thick Placenta in Pregnancy: A Review,” Obstetrical and Gynecological Survey 77, no. 9 (2022): 547–557, 10.1097/OGX.0000000000001051. 36136077

[6] B. A. Burnett , C. M. Parobek , M. A. Shanahan , et al., “Prenatal Sonographic Predictors of Maternal Mirror Syndrome in Hydropic Fetuses,” American Journal of Obstetrics & Gynecology MFM 7, no. 12 (2025): 101787, 10.1016/j.ajogmf.2025.101787. 40983242

[7] S. Allarakia , H. A. Khayat , M. M. Karami , et al., “Characteristics and Management of Mirror Syndrome: A Systematic Review (1956–2016),” Journal of Perinatal Medicine 45, no. 9 (2017), 10.1515/jpm-2016-0422. 28315852

[8] S. Biswas , J. Gomez , R. Horgan , et al., “Mirror Syndrome: A Systematic Literature Review,” American Journal of Obstetrics & Gynecology MFM 5, no. 9 (2023): 101067, 10.1016/j.ajogmf.2023.101067. 37385374

[9] Z. Han , X. Chen , Q. Wang , et al., “Clinical Characteristics and Risk Factors of Mirror Syndrome: A Retrospective Case‐Control Study,” BMC Pregnancy and Childbirth 21, no. 1 (2021): 660, 10.1186/s12884-021-04143-3. 34583666 PMC8480018

[10] J. Espinoza , R. Romero , J. K. Nien , et al., “A Role of the Anti‐Angiogenic Factor sVEGFR‐1 in the ‘Mirror Syndrome’ (Ballantyne’s Syndrome),” Journal of Maternal‐Fetal and Neonatal Medicine 19, no. 10 (2006): 607–613, 10.1080/14767050600922677. 17118734 PMC6941849

[11] H. Chai , Q. Fang , X. Huang , Y. Zhou , and Y. Luo , “Prenatal Management and Outcomes in Mirror Syndrome Associated With Twin–Twin Transfusion Syndrome,” Prenatal Diagnosis 34, no. 12 (2014): 1213–1218, 10.1002/pd.4458. 25043377

[12] R. Chen , M. Liu , J. Yan , et al., “Clinical Characteristics of Mirror Syndrome: A Retrospective Study of 16 Cases,” Journal of Obstetrics and Gynaecology 41, no. 1 (2021): 73–76, 10.1080/01443615.2020.1718621. 32420780

[13] Y. Zhao , G. Liu , J. Wang , J. Yang , D. Shen , and X. Zhang , “Mirror Syndrome in a Chinese Hospital: Diverse Causes and Maternal Fetal Features,” Journal of Maternal‐Fetal and Neonatal Medicine 26, no. 3 (2013): 254–258, 10.3109/14767058.2012.733765. 23035798

[14] G. Hirata , S. Aoki , K. Sakamaki , T. Takahashi , F. Hirahara , and H. Ishikawa , “Clinical Characteristics of Mirror Syndrome: A Comparison of 10 Cases of Mirror Syndrome With Non‐Mirror Syndrome Fetal Hydrops Cases,” Journal of Maternal‐Fetal and Neonatal Medicine (October 2015): 1–5, 10.3109/14767058.2015.1095880. 26482778

[15] H. Mogharbel , J. Hunt , R. D’Souza , and S. R. Hobson , “Clinical Presentation and maternal‐fetal Outcomes of Mirror Syndrome: A Case Series of 10 Affected Pregnancies,” Obstetric Medicine 15, no. 3 (2022): 190–194, 10.1177/1753495X211058043. 36262819 PMC9574449

[16] T. Braun , M. Brauer , I. Fuchs , et al., “Mirror Syndrome: A Systematic Review of Fetal Associated Conditions, Maternal Presentation and Perinatal Outcome,” Fetal Diagnosis and Therapy 27, no. 4 (2010): 191–203, 10.1159/000305096. 20357423

[17] A. Gedikbasi , K. Oztarhan , Z. Gunenc , et al., “Preeclampsia due to Fetal Non‐Immune Hydrops: Mirror Syndrome and Review of Literature,” Hypertension in Pregnancy 30, no. 3 (2011): 322–330, 10.3109/10641950903323244. 21174577

[18] F. El Mangoub , R. A. Bouhou , Z. Idri , J. Kouach , K. Guelzim , and D. M. Rahali , “Syndrome de Ballantyne compliqué d’eclampsie: À propos d’un cas et revue de la littérature,” Pan African Medical Journal 30 (2018), 10.11604/pamj.2018.30.238.15296. PMC6295295 30574257

[19] A. Giacobbe , R. Grasso , M. L. Interdonato , et al., “An Unusual Form of Mirror Syndrome: A Case Report,” Journal of Maternal‐Fetal and Neonatal Medicine 26, no. 3 (2013): 313–315, 10.3109/14767058.2012.722734. 22957971

[20] D. T. O’Driscoll , “A Fluid Retention Syndrome Associated With Severe Iso‐Immunization to the Rhesus Factor,” BJOG: An International Journal of Obstetrics & Gynaecology 63, no. 3 (1956): 372–374, 10.1111/j.1471-0528.1956.tb05502.x. 13332452

[21] M. E. Norton , S. P. Chauhan , and J. S. Dashe , “Society for Maternal‐Fetal Medicine (SMFM) Clinical Guideline #7: Nonimmune Hydrops Fetalis,” American Journal of Obstetrics and Gynecology 212, no. 2 (2015): 127–139, 10.1016/j.ajog.2014.12.018. 25557883

[22] M. Schoberer , A. Rink , W. Rath , and T. Orlikowsky , “Ballantyne Syndrome and Congenital Anaemia Associated With Parvovirus B19 Infection: Case Report and Review,” Zeitschrift für Geburtshilfe und Neonatologie 217, no. 5 (2013): 183–188, 10.1055/s-0033-1355349. 24170444

[23] P. T. Wu , K. L. Huang , C. C. Tsai , H. H. Cheng , Y. J. Lai , and T. Y. Hsu , “A Singleton Pregnancy With Placental Chorioangioma and Hydrops Fetalis Complicated With Mirror Syndrome and Ritodrine‐Induced Side Effects: A Case Report,” BMC Pregnancy and Childbirth 24, no. 1 (2024): 213, 10.1186/s12884-024-06391-5. 38509456 PMC10956381

[24] A. S. Pais , A. L. F. A. D. Areia , S. M. P. Franco , E. M. F. Fonseca , and J. P. A. S. Moura , “Mirror Syndrome Assocciated With Patau Syndrome: A Case Report,” Revista Brasileira de Ginecologia e Obstetrícia 40, no. 7 (2018): 430–432, 10.1055/s-0038-1653975. 29768639

[25] T. Van Mieghem , A. Al‐Ibrahim , J. Deprest , et al., “Minimally Invasive Therapy for Fetal Sacrococcygeal Teratoma: Case Series and Systematic Review of the Literature,” Ultrasound in Obstetrics and Gynecology 43, no. 6 (2014): 611–619, 10.1002/uog.13315. 24488859

[26] S. Ren , T. Bhavsar , and J. Wurzel , “CHAOS in the Mirror: Ballantyne (Mirror) Syndrome Related to Congenital High Upper Airway Obstruction Syndrome,” Fetal and Pediatric Pathology 31, no. 6 (2012): 360–364, 10.3109/15513815.2012.659400. 22468720

[27] Y. Katoh , T. Seyama , N. Mimura , et al., “Elevation of Maternal Serum sFlt‐1 in Pregnancy With Mirror Syndrome Caused by Fetal Cardiac Failure,” Oxford Medical Case Reports 2018, no. 3 (2018), 10.1093/omcr/omx112. PMC6007303 29942527

[28] J. Y. Lee and J. Y. Hwang , “Mirror Syndrome Associated With Fetal Leukemia,” Journal of Obstetrics and Gynaecology Research 41, no. 6 (2015): 971–974, 10.1111/jog.12654. 25546294

[29] R. Chiaki , Y. Takahashi , T. Shiga , et al., “P 21.11: Decrease of Human Chorionic Gonadotropin (hCG) in the Cases of Fetal Pleural Effusion Following Effective Thoraco‐Amniotic Shunting (TAS),” supplement, Ultrasound in Obstetrics and Gynecology 44, no. S1 (2014): 312–313, 10.1002/uog.14420.

[30] M. Matsubara , M. Nakata , S. Murata , I. Miwa , M. Sumie , and N. Sugino , “Resolution of Mirror Syndrome After Successful Fetoscopic Laser Photocoagulation of Communicating Placental Vessels in Severe Twin–Twin Transfusion Syndrome,” Prenatal Diagnosis 28, no. 12 (2008): 1167–1168, 10.1002/pd.2128. 18925575

[31] B. Halim , H. P. Lubis , and T. Amalia , “Ovarian Hyperstimulation Syndrome in Spontaneous Pregnancy With Sacrococcygeal Teratoma Complicated by Maternal Mirror Syndrome: A Comorbidity,” Middle East Fertility Society Journal 21, no. 4 (2016): 277–280, 10.1016/j.mefs.2016.05.002.

[32] N. Mehandru , J. Harris , O. Kalinkin , and A. Liguori , “Hyperreactio Luteinalis in the Third Trimester Complicating Fetal Hydrops and Mirror Syndrome,” Journal of Clinical Gynecology & Obstetrics 6, no. 2 (2017): 41–44, 10.14740/jcgo439w.

[33] R. B. Gherman , M. H. Incerpi , D. A. Wing , and T. M. Goodwin , “Ballantyne Syndrome: Is Placental Ischemia the Etiology?,” Journal of Maternal‐Fetal Medicine 7, no. 5 (1998): 227–229, 10.1002/(SICI)1520-6661(199809/10)7:5<227::AID-MFM3>3.0.CO;2-I. 9775990

[34] J. C. Livingston , K. M. Malik , T. M. Crombleholme , F. Y. Lim , and B. M. Sibai , “Mirror Syndrome: A Novel Approach to Therapy With Fetal Peritoneal–Amniotic Shunt,” Obstetrics & Gynecology 110, no. 2 (2007): 540–543, 10.1097/01.AOG.0000275259.03301.b2. 17666658

[35] A. Chimenea , L. García‐Díaz , A. M. Calderón , M. M. D. L. Heras , and G. Antiñolo , “Resolution of Maternal Mirror Syndrome After Succesful Fetal Intrauterine Therapy: A Case Series,” BMC Pregnancy and Childbirth 18, no. 1 (2018): 85, 10.1186/s12884-018-1718-0. 29625560 PMC5889608

[36] N. R. Gavin , A. D. Forrest , M. Rosner , J. L. Miller , and A. A. Baschat , “The Role of Fetal Therapy in the Management of Mirror Syndrome: A Narrative Review,” Journal of Maternal‐Fetal and Neonatal Medicine 37, no. 1 (2024): 2345307, 10.1080/14767058.2024.2345307. 38679585

[37] K. Deurloo , R. Devlieger , and D. Oepkes , “Maternal Hydrops Syndrome Following Successful Treatment of Fetal Hydrops by Shunting of Bilateral Hydrothorax,” Prenatal Diagnosis 23, no. 11 (2003): 944–945, 10.1002/pd.729. 14634984

